# Norovirus Outbreak Associated with Swimming in a Recreational Lake Not Influenced by External Human Fecal Sources in The Netherlands, August 2012

**DOI:** 10.3390/ijerph15112550

**Published:** 2018-11-14

**Authors:** Franciska M. Schets, Harold H. J. L. van den Berg, Harry Vennema, Manon T. M. Pelgrim, Cees Collé, Saskia A. Rutjes, Willemijn J. Lodder

**Affiliations:** 1 National Institute for Public Health and the Environment, P.O. Box 1, 3720 BA Bilthoven, The Netherlands; harold.van.den.berg@rivm.nl (H.H.J.L.v.d.B.); harry.vennema@rivm.nl (H.V.); saskia.rutjes@rivm.nl (S.A.R.); willemijn.lodder@rivm.nl (W.J.L.); 2 Public Health Service Veiligheids-en Gezondheidsregio Gelderland-Midden, Postbus 5364, 6802 EJ Arnhem, The Netherlands; manon.pelgrim@vggm.nl; 3 Province of Gelderland, Postbus 9090, 6800 GX Arnhem, The Netherlands; c.colle@gelderland.nl

**Keywords:** outbreak, recreational water, norovirus, swimming

## Abstract

Swimming in fecally contaminated recreational water may lead to gastrointestinal illness. A recreational water-associated outbreak of norovirus (NoV) infections affecting at least 100 people in The Netherlands occurred in August 2012. Questionnaire responses from patients indicated swimming in recreational lake Zeumeren as the most likely cause of illness. Most patients visited the lake during the weekend of 18–19 August, during which the weather was exceptionally warm (maximum temperatures 32–33 °C), and visitor numbers elevated. Patients, mostly children, became ill with gastroenteritis 1–6 days (median 2 days) after exposure. Four stool samples from patients were NoV GI positive. Subsurface sandy soil from one of the beaches where most patients swam was NoV GI positive; the water sample was negative. The epidemiological curve and the timeline of investigation based on reported symptoms demonstrate the difficulty in discovering the source in recreational water outbreaks. A NoV outbreak in a recreational lake that is not subjected to external fecal contamination sources shows the need for active communication about human shedding of viruses during and after diarrheal episodes and the advice to refrain from swimming, even a few weeks after the symptoms have resolved.

## 1. Introduction

Noroviruses (NoV) are the leading cause of diarrhea in all age groups worldwide and they are primarily transmitted through the fecal-oral route [1]. The viruses are extremely contagious, with an estimated 50% infectious dose (ID_50_) as low as 2.6 (aggregated) viral particles [2]. Primary cases often result from exposure to contaminated food or water, whereas person-to-person contact results in further spread of the infection. Infected persons shed large numbers of viral particles into the environment, and (low level) shedding may continue after symptoms have resolved [1,3]. Symptoms of NoV infections are mainly projectile vomiting and watery non-bloody diarrhoea, but fever, abdominal cramping and nausea do also occur. The incubation period is 10 to 51 h and illness is generally self-limiting, normally lasting 2–3 days [1,4], but the duration of illness can be prolonged to 4–6 days in children younger than 11 years of age [5].

NoV have been genetically classified into five genogroups (G) [6]. GI and GII are primarily associated with human disease [1]. The GII.4 cluster is responsible for most human norovirus outbreaks [7]; for example, over 80% of the outbreaks in the United States (1994–2006) were caused by strains from this cluster [4]. GI strains are more often associated with waterborne outbreaks than other genogroups [8]. NoV have been the cause of outbreaks related to contaminated municipal water supplies, e.g., in Sweden [9], drinking water wells, e.g., in The Netherlands [10], ground water, e.g., in South Korea [11], and various recreational water settings [12], including a fountain in Belgium, where Dutch school children became ill during a school trip [13], a swimming pool [14] and a fresh water lake in the United States [15], and a recreational lake in Finland [16]. A survey of recreational water associated viral disease outbreaks, occurring between 1977 and 2006, identified noroviruses as the cause of the illness in 45% (*n* = 25) of the outbreaks [12]. The majority of these outbreaks resulted from exposure to lakes (56%, *n* = 14). In a recently published study of bathing water related outbreaks in Finland that all occurred in 2014, epidemiological and microbiological data showed that NoV was the main causative agent [17].

This paper describes the occurrence and investigation of a recreational water associated outbreak of NoV infections affecting at least 100 people in The Netherlands in August 2012.

## 2. Materials and Methods

### 2.1. Outbreak Description

**Figure 1:**
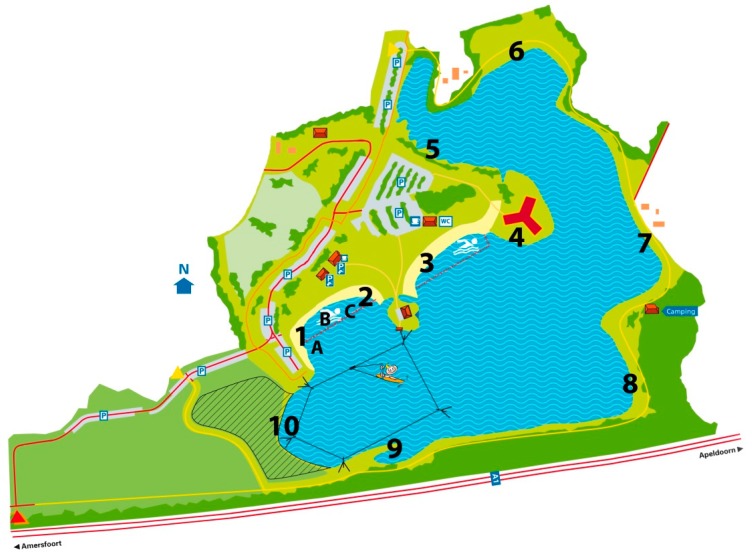
Recreational water lake Zeumeren, The Netherlands, with beaches 1*—*10 and sampling points. A*—*C. Beaches 1*—*2 and 3 are the official beaches, which are sampled for monitoring according to the EU BWD [18]. Sampling points for the outbreak investigation: A*—*water and subsurface sandy soil sample taken, B and C*—*subsurface sandy soil samples taken.

On Monday, 20 August 2012, over 20 people reported gastro-intestinal complaints to the province of Gelderland after swimming in recreational lake Zeumeren in Barneveld, The Netherlands. During the week of 20–24 August, over 100 people reported the same with Public Health Service Veiligheids-en Gezondheidsregio Gelderland-Midden (VGGM). All patients, who were mainly children, had visited the lake during the previous weekend of 18 and 19 August. During that weekend, the weather in The Netherlands was exceptionally warm with maximum temperatures of 32–33 °C (at weather station De Bilt, the station closest to Zeumeren) (www.knmi.nl). Because of the warm weather, several thousands of people swam at Zeumeren during the weekend. Most patients became ill one to one-and-a-half day after exposure to the lake water, and had swum at two of the ten possible beaches surrounding the lake (Figure 1). The majority of the patients reported short-term symptoms and recovered rapidly.

**Figure 2:**
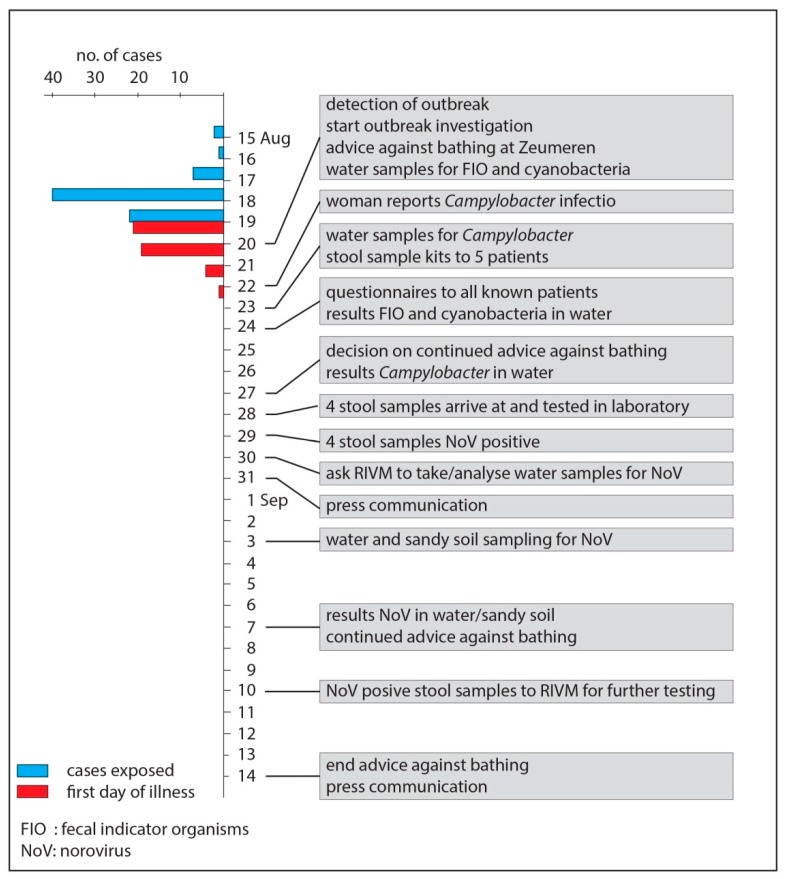
Epidemiological curve, indicating the days on which the cases (*n*_total_ = 45) were exposed and their first day of illness, and the timeline of the outbreak investigation.

Investigation of the outbreak started on Monday, 20 August 2012; as a precaution, an advice against bathing in lake Zeumeren was set at the end of the afternoon on that same day. The advice against bathing was removed on 14 September 2012 (Figure 2). Since occasionally swimmers were observed in the lake after setting the advice against bathing, the case definition was defined as: all people that visited lake Zeumeren between 15 August and 24 August 2012, and subsequently developed gastro-intestinal symptoms.

### 2.2. Site Description

Lake Zeumeren is located in a rural area (community Barneveld) in the center of The Netherlands. It is a 32-hectare isolated fresh water lake with stagnant water, with groundwater and rainwater as sources. The maximum water depth is 17 m and it has a sandy soil. Lake Zeumeren is an official bathing site, where the water quality is monitored for fecal indicator parameters *Escherichia coli* and intestinal enterococci, according to the European Bathing Water Directive (EU BWD) [18] with a fortnightly frequency during the bathing season (1 May–1 October). The lake is surrounded by beaches, two of which are identified as the official beaches, with demarcated bathing areas. At the two official beaches, regular samples are taken for monitoring according to the EU BWD (Figure 1). The bathing zone has a surface of 1.7 hectare, a maximum water depth of 1.5 m, and a total beach length of 530 m. On-site facilities include toilets, showers, waste bins, and play equipment; the play equipment is located on the dry land of beach 4 (Figure 1). Dogs are not allowed at the location during the bathing season. The bathing water profile indicates that there is no connection to other water bodies, and that the water quality is not influenced by wastewater treatment plants or run-off from agricultural land. A substantial seagull population foraging at the lake may however occasionally influence the water quality. According to the EU BWD, water quality at lake Zeumeren is classified as ‘excellent’.

### 2.3. Epidemiological Investigation

The province of Gelderland and the Public Health Service VGGM sent questionnaires by email on 24 August 2012 to all patients with whom they had primary contact and for whom they had kept records (*n* = 36). The patients were asked to answer the questions and to forward the questionnaire to anyone they knew who had swum at the same site during the same period and had become ill with the same symptoms. This approach was chosen in an attempt to rapidly identify the source of the outbreak. Since there was no aim at performing a full case-control study, no matching controls were sought. By allowing questionnaires to be forwarded, no records were (or could be) kept of the number of questionnaires distributed. Apart from demographic questions about age and gender, questionnaires included information requests about having visited lake Zeumeren (date between 15 August and 24 August 2012), having entered the water (including specification of beaches visited), having swum (including duration of swimming), health conditions (choose from diarrhea, vomiting, fever, fatigue, skin conditions, other), using the on-site toilets, and having bought and consumed food and beverages on-site. Analysis of questionnaire responses was done by using SPSS (IBM, Armonk, NY, USA, version 22).

### 2.4. Microbiological Investigation of Clinical Samples

Five patients were asked to send in stool samples; samples from four patients were received and analyzed for the bacterial enteropathogens *Salmonella*, *Shigella*, *Campylobacter*, shiga toxin-producing *Escherichia coli* (STEC) and *Yersinia*, and NoV at the medical microbiological laboratory of the Rijnstate Hospital in Arnhem, The Netherlands, by using routine PCR. Later, the four samples were sent to the National Institute for Public Health and the Environment (RIVM) for further testing. At RIVM, the samples were analyzed for the presence of NoV by using a real-time RT-PCR assay, with primers, probes and PCR conditions as previously described [19].

### 2.5. Environmental Investigation

In order to narrow down the outbreak and to identify the source, several leads were followed. In response to the reported cases of gastroenteritis, water samples were taken from lake Zeumeren at the two official beaches (Figure 1) on 20 August 2012 and were analyzed for fecal indicator parameters *E. coli* and intestinal enterococci, by using the most probable number methods specified in the EU BWD [18]. 

Although lake Zeumeren does not have a history of problems with cyanobacteria, the presence of a faint greenish colouring of the water led to visual inspection for the presence of cyanobacteria and sampling of the water at beach 4 (Figure 1) for determination of the dominant cyanobacterium genus by microscopy and toxin levels by ELISA on Monday, 20 August 2012.

On Wednesday, 22 August, a woman reported to the province of Gelderland having fallen ill after swimming in lake Zeumeren during the weekend prior to the outbreak weekend (11–12 August). She attended a physician and had blood, urine and feces examined, which revealed a *Campylobacter jejuni* infection that was treated with antibiotics. Although the woman did not meet the case definition, it was decided to follow this lead because of the known nuisance caused by gulls at lake Zeumeren that take over the beaches after the bathers have left. Water samples were taken at the two official beaches on 23 August and analyzed for the presence of *Campylobacter* spp. by using a membrane filtration method with incubation on Karmali Agar at 42 ± 0.5 °C for 48 ± 2 h (in house method of Het Waterlaboratorium, Haarlem, The Netherlands). 

When the four analyzed stool samples appeared to be NoV positive, RIVM was contacted on 30 August and asked to take environmental samples for NoV analysis at lake Zeumeren. Sampling was done on 3 September 2012. Water and subsurface sandy soil samples were only taken from the beaches where most of the patients had stayed or swum during the weekend of 18 and 19 August as was pointed out by the questionnaire results. One water sample was taken at sampling point A, and one subsurface sandy soil sample was taken at each of the sampling points A–C (Figure 1).

The water sample (600 L) was taken by using an on-site filtration adsorption-elution procedure for concentration of the sample [20,21]. The eluate was further concentrated by ultrafiltration under high pressure (three bars) using a cellulose-acetate filter with a nominal molecular weight limit of 10,000 (Sartorius). The ultrafilter was rinsed with 3% beef extract (pH 9.0) resulting in the final concentrate of approximately 40 mL which was stored at −70°C until further analysis. RNA extraction was performed on 0.2, 1 and 5 mL of the concentrate as described by Rutjes et al. [21].

Subsurface sandy soil samples of approximately 50 g were taken by using a tubular soil sampler. Subsequently, 10 mL 0.05 M glycine buffer pH 9.0 was added to 5.0 g of each sandy soil sample and mixed at 500 rpm for 35 min at 4°C, after which it was allowed to settle for 10 min (in house method RIVM). RNA extraction was done according to Rutjes et al. [21], by adding 20 and then 9 mL of lysis buffer (Biomerieux, Boxtel, The Netherlands) to 5 mL and 1 mL volumes, respectively, of the supernatant. Purification of the RNA extract was done by using a Qiagen RNeasy kit according to the manufacturers’ instructions.

A real time reverse transcriptase PCR (RT-PCR) assay as described by Verhaelen et al. [19] was performed to detect the presence of norovirus GI and GII RNA. The sequence of the detected norovirus strain was determined by directly sequencing the purified RT-PCR product, which was 86 nucleotides long.

## 3. Results

### 3.1. Epidemiological Investigation

Public Health Service VGGM received 45 filled-in questionnaires that met the case definition. Of the respondents, 56% (*n* = 25) were female. The age of the respondents ranged between 0 and 59 years of age, with more than half being children of 0–12 years of age (53%, *n* = 24), while the others were adults of 18–59 years of age (47%, *n* = 21). All respondents visited lake Zeumeren between 15 and 19 August, but the majority were at the site on 18 August (89%, *n* = 40) and/or 19 August (49%, *n* = 22). In total, 27 cases were exposed on more than one day during the specified period. All cases went into the water for swimming and became ill afterwards, with health complaints starting 1–6 days (average and median 2 days) after exposure to the bathing water and beaches at Lake Zeumeren and lasting for 0.5–6 days (average and median 2 days). Reported health conditions were (multiple answers per case allowed): diarrhea (84%, *n* = 38), vomiting (82%, *n* = 37), fatigue (49%, *n* = 22), and fever (16%, *n* = 7). None of the cases reported skin conditions, and eight cases reported other complaints than those indicated above, of which seven were related to gastro-intestinal discomfort. One case reported a headache.

Forty-four of the cases indicated at which beach they had swum (multiple answers per case were allowed), showing that most cases swam at beach 1 (70%, *n* = 31) and/or beach 2 (32%, *n* = 14). Most cases (82%, *n* = 37) were in the water for 30 min to over an hour.

Fifty-eight percent (*n* = 26) of the cases used the permanent on-site toilets, the others did not. Buying and consuming on-site sold food and beverages were not very common: 87% (*n* = 39) did not buy or consume food and 98% (*n* = 44) did not buy or consume beverages.

### 3.2. Microbiological Investigation of Clinical Samples

The four investigated stool samples were positive with NoV GI, and not with any of the other aetiologies. The fecal samples contained NoV genotype GI.Pd-GI.3. The partial genomic sequence of the strain was submitted to Genbank under accession number MH828423. The size of the PCR product was 1116 base pairs, including the primers. Sequence analysis yielded 1007 nucleotides, 733 overlapping with the typing region of open reading frame 1 (ORF1), and 275 overlapping with the typing region of ORF2. Genotype assignment by phylogenetic analysis was supported by 100% and 99% bootstrap analysis, for ORF1 and ORF2, respectively. Typing from RT-PCR products is reliable if the fragment has >100 nucleotides overlapping with both of the typing regions of ORF1 and ORF2.

### 3.3. Environmental Investigation

Water samples taken on 20 August 2012 showed levels of *E. coli* at the official beaches of 16 most probable number (mpn)/100 mL at beach 1–2 and 6 mpn/100 mL at beach 3. Compared to previous and later routine samplings during the same bathing season, slightly elevated levels of intestinal enterococci at both beaches, 230 mpn/100 mL and 160 mpn/100 mL, respectively, were observed.

Examination of the samples taken for cyanobacteria analyses by microscopy demonstrated the presence of *Microcystis* spp. at beach 4; intracellular microcystin levels were very low (<2 µg/L).

*Campylobacter* spp. were present in the water samples taken at the official beaches (one sample per beach) on 23 August. Numbers appeared to be low, but could not be exactly determined due to overgrowth of the culture medium. Further identification to the species level was not done.

NoV GI was detected in the subsurface sandy soil sample taken at sampling point A (beach 1). The other subsurface sandy soil samples and the water sample were NoV negative. Sequencing of the RT-PCR product from the subsurface sandy soil sample was impossible because the method for typing of RT-PCR products is less sensitive than the detection method, and the amount of RNA in the subsurface sandy soil sample appeared to be too low.

## 4. Discussion

Following the reporting of many cases of gastroenteritis that seemed related to exposure to a recreational lake in The Netherlands during an exceptionally warm weekend in August 2012, several leads were investigated in order to discover the etiological agent and the source of the outbreak. Although the epidemiological investigation implicated the recreational lake as the most likely location of contracting illness, the different directions in which the investigation went, based on reported symptoms, demonstrate the difficulty in narrowing down the possible cause of recreational water related outbreaks. The results of the examination of patient material can ease the search for the etiological agent and the source; however, in this case, the reporting of a patient with a *Campylobacter* infection in combination with the presence of a large seagull population resulted in choosing, what later appeared to be, the wrong direction. Following several leads and having various patient and environmental samples examined takes time, and in this case, led to the examination of water and subsurface sandy soil samples only over two weeks after the outbreak presumably started, thus diminishing the chances of detecting the etiological agent. Due to of the time gap between the onset of the outbreak and the environmental sampling, subsurface sandy soil sampling was performed in addition to the normal procedure of water sampling because sedimentation of virus particles during the delay period could have resulted in the presence of virus particles in the subsurface sandy soil [22]. In this outbreak investigation, this appeared to be true and therefore subsurface sandy soil sampling could be considered while environmental sampling in outbreak situations is delayed. However, even if environmental samples are taken sooner, factors such as dispersion, dilution, sedimentation and inactivation of the microorganisms in the water body may result in the inability to detect the microorganisms that caused the outbreak [23]. The detection of NoV GI in the stool samples of outbreak related patients and the detection of NoV GI in the subsurface sandy soil of the beach where most patients swam support the probability that exposure to the lake resulted in the outbreak. NoV G I strains have a stronger association with waterborne transmission than GII strains [8,24], possibly because they are more stable in water than GII strains [8]. In a survey of recreational waters in Europe, 9.4% of 1440 samples were positive with NoV GI and GII [25], thus demonstrating the presence of NoV in randomly selected recreational waters.

The epidemiological investigation showed that most of the patients were children. It has been demonstrated that, while swimming, compared to adults, children spend more time in the water and ingest a larger volume of water, thus increasing their exposure [26,27] and resulting in a higher infection risk and attributable disease burden [28]. High exposure of children in combination with the low infectious dose of NoV [2] and the incompletely developed immune system in young children explain the high number of affected children. A possible bias is that parents are more likely to report health complaints and sooner when their children are involved.

The detection of the same NoV genogroup in subsurface sandy soil and in patients two weeks after the onset of the outbreak shows that NoV may be present in the environment for prolonged time through accumulation in sediment [22], and may even be re-dispersed when bathers whirl the soil. Although only RNA from virus particles was detected and no information about their infectivity was available, these results indicate the value of a prolonged advice against bathing. Lake Zeumeren is an isolated lake that is not subject to external sources of fecal contamination that may introduce NoV, such as sewage water treatment plants, and the majority of the cases did not buy or consume on-site sold food and beverages. Therefore, a food-related cause of the outbreak is unlikely and the most plausible cause of contamination of the water is an infected human. Evidence of a single person being responsible for contamination of the water was, however, not discovered, as is often the case [8,24]. Prevention of outbreaks that have an infected human as the source can be done by active communication of the long-standing advice of not to swim while having diarrhea, combined with the information that viral shedding continues even after symptoms have resolved, although this does not prevent occasional asymptomatic shedding [29].

## 5. Conclusions

An outbreak of gastrointestinal illness among over 100 visitors of a recreational lake in The Netherlands during an exceptionally warm weekend was caused by NoV. The results of the epidemiological investigation, combined with the detection of NoV GI in the stool samples of outbreak related patients and in the subsurface sandy soil of the beach where most patients swam, suggest that exposure to the recreational lake resulted in the outbreak. Although the primary contamination source has not been discovered, the most likely cause of contamination of the water is an infected human, because the lake is an isolated water body that is not subject to external sources of fecal contamination. An outbreak like this warrants active communication about human shedding of viruses during and after diarrheal episodes and the advice to refrain from swimming. The epidemiological curve and the timeline of investigation based on reported symptoms demonstrate the difficulty in discovering the source in recreational water outbreaks.
